# Protein Kinase D Isoforms Differentially Modulate Cofilin-Driven Directed Cell Migration

**DOI:** 10.1371/journal.pone.0098090

**Published:** 2014-05-19

**Authors:** Heike Döppler, Ligia I. Bastea, Sahra Borges, Samantha J. Spratley, Sarah E. Pearce, Peter Storz

**Affiliations:** Department of Cancer Biology, Mayo Clinic, Jacksonville, Florida, United States of America; King’s College London, United Kingdom

## Abstract

**Background:**

Protein kinase D (PKD) enzymes regulate cofilin-driven actin reorganization and directed cell migration through both p21-activated kinase 4 (PAK4) and the phosphatase slingshot 1L (SSH1L). The relative contributions of different endogenous PKD isoforms to both signaling pathways have not been elucidated, sufficiently.

**Methodology/Principal Findings:**

We here analyzed two cell lines (HeLa and MDA-MB-468) that express the subtypes protein kinase D2 (PKD2) and protein kinase D3 (PKD3). We show that under normal growth conditions both isoforms can form a complex, in which PKD3 is basally-active and PKD2 is inactive. Basal activity of PKD3 mediates PAK4 activity and downstream signaling, but does not significantly inhibit SSH1L. This signaling constellation was required for facilitating directed cell migration. Activation of PKD2 and further increase of PKD3 activity leads to additional phosphorylation and inhibition of endogenous SSH1L. Net effect is a dramatic increase in phospho-cofilin and a decrease in cell migration, since now both PAK4 and SSH1L are regulated by the active PKD2/PKD3 complex.

**Conclusions/Significance:**

Our data suggest that PKD complexes provide an interface for both cofilin regulatory pathways. Dependent on the activity of involved PKD enzymes signaling can be balanced to guarantee a functional cofilin activity cycle and increase cell migration, or imbalanced to decrease cell migration. Our data also provide an explanation of how PKD isoforms mediate different effects on directed cell migration.

## Introduction

In order to migrate towards a chemotactic stimulus cells activate cofilin at the leading edge [1], [2], [3]. Once released from the membrane, cofilin is active and severs F-actin structures. Cofilin activity is regulated by phosphorylation at serine residue S3 [4]. Phosphorylation of S3 leads to cofilin inactivation and is mediated by LIM domain kinase (LIMK), whereas the phosphatase slingshot 1L (SSH1L) dephosphorylates this site [3]. Both regulatory enzymes, LIMK and SSH1L guarantee a functional cofilin activity cycle (cyclic activation and reactivation of cofilin to facilitate F-actin reorganization processes) at the leading edge of cells [5]. Tipping the balance of activities of these enzymes all results in imbalance of the cofilin activity cycle and a decrease in cell migration [6], [7].

The protein kinase D (PKD) family of serine/threonine kinases consists of three isoforms, PKD1, PKD2 and PKD3 [8]. While PKD1 and PKD2 share high homology in their structure, PKD3 lacks some regulatory elements, for instance a PDZ binding motif [9] and a phosphorylation motif for Src family kinases [10]. Consequently, PKD1 and PKD2 show more redundancy in their functions. PKD enzymes have been implicated in regulating directed cell migration either by controlling anterograde membrane trafficking [11], or by directly impacting F-actin reorganization processes at the leading edge [12]. Multiple substrates for PKD have been identified at the leading edge, all of which can contribute to directed cell migration. These include cortactin [13], Evl-1 and VASP [14], [15] and several others (summarized in [16]). In addition to this, PKD enzymes regulate cofilin activity through modulating its phosphorylation status [17]. For example, PKD isoforms have been shown to phosphorylate SSH1L at S978, and this leads to its inactivation, binding to 14-3-3 proteins and localization to the cytosol [17], [18], [19]. PKD isoforms also can phosphorylate and activate PAK4, an upstream kinase for LIMK1/2. Inactivation of SSH1L as well as activation of the PAK4/LIMK pathway by PKD can dramatically increase phospho-S3-cofilin levels within cells, resulting in a dysfunctional cofilin activity cycle, decreased F-actin free barbed end formation, and a decrease in directed cell migration [19], [20]. While the expression of constitutively-active alleles of all PKD isoforms decreases in cell migration [19], it was also shown that treatment of cells with pan PKD inhibitors decreases directed cell migration [21], [22]. These contradictory results may be dependent on the cellular signaling context, namely the activity status of PAK4/LIMK or SSH1L. Moreover, endogenous PKD activity levels under normal growth conditions could be relevant. Modulation of expression or activity of each of these components may tip the balance towards a non-functional cofilin activity cycle, either by mediating cofilin hyper- or hypo-phosphorylation, with a net effect of decreased migration under both conditions. Consequently, endogenous expression levels and activity of PKD isoforms, as well as cofilin regulatory pathways need to be determined for each cellular system, before general conclusions can be drawn.

Goal of this study was to determine if the different PKD isoforms regulate directed cell migration by having differential effects on cofilin regulatory pathways. To determine this, we utilized two cell lines (HeLa and MDA-MB-468) that express only the subtypes PKD2 and PKD3. We show that both PKD subtypes can form a complex in which PKD3 under normal growth conditions is basally active and mediates PAK4/LIMK activity. Under the same conditions SSH1L was unphosphorylated (and active). Consequently, a functional cofilin activity cycle was guaranteed. Unlike PKD3, PKD2 was not basally active under normal growth conditions. In response to a stimulus (active RhoA) PKD2 was activated. When a constitutively-active (CA) allele of PKD2 was expressed to mimic full activation, phospho-S3-coflin levels increased dramatically since SSH1L was inactivated by phosphorylation. This resulted in decreased cell migration. Taken together, our data suggest that complexes of PKD enzymes differentially regulate the delicate balance of phosphorylation and dephosphorylation events that facilitate a functional cofilin activity cycle.

## Results

### PKD2 and PKD3 can form a Complex, in which PKD3 shows Basal Activity

**Figure 1:**
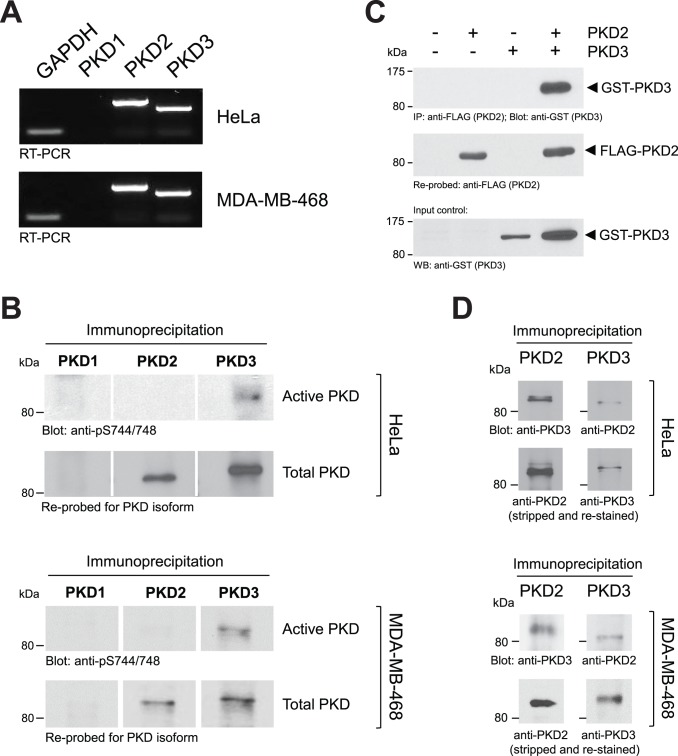
In HeLa cells PKD3 is basally active and forms a complex with PKD2. **A:** HeLa or MDA-MB-468 cells were analyzed for expression of PKD1, PKD2, PKD3 and GADPH using RT-PCR. **B:** Endogenous PKD1, PKD2 or PKD3 were immunoprecipitated from HeLa or MDA-MB-468 cells using isoform-specific antibodies (all antibodies were tested for cross-reactivity between isoforms and were isoform specific – not shown). Samples were subjected to SDS-PAGE and analyzed for PKD activity by immunoblotting with anti-pS744/748 antibody (recognizes the phosphorylated activation loop of all three isoforms). Samples were then re-probed for the respective isoform (anti-PKD1, anti-PKD2 or anti-PKD3 antibodies). **C:** HeLa cells were transfected with FLAG-tagged PKD2 and GST-tagged PKD3 or vector controls as indicated. PKD2 was immunoprecipitated (anti-FLAG), samples separated on SDS-PAGE, transferred to nitrocellulose and then probed for co-immunoprecipitated PKD3 (anti-GST). Nitrocellulose then was stripped and re-probed for PKD2 (anti-FLAG). PKD3 input was controlled by Western blotting of lysates against GST-PKD3 (anti-GST). **D:** Endogenous PKD2 was immunoprecipitated from HeLa or MDA-MB-468 cells and after SDS-PAGE samples were analyzed for co-immunoprecipitated PKD3; or *vice versa*. Experiments shown in A-D were performed at least three times with similar results.

HeLa and MDA-MB-468 cells under normal growth conditions mainly express mRNA encoding the PKD isoforms PKD2 and PKD3 (**Fig. 1A**). Results obtained with RT-PCR were confirmed by immunoprecipitations of endogenous PKD1, PKD2 or PKD3 using subtype-specific antibodies (**Fig. 1B**, bottom panels). Moreover, in both cell lines under normal growth conditions, PKD3 showed basal activity, whereas PKD2 was not basally active (**Fig. 1B**, top panels). Activity was judged by staining of the immunoprecipitates for phosphorylation at the activation loop serines (S706 and S710 for PKD2; S731 and S735 for PKD3) using an antibody (anti-pS744/748) that detects activation loop phosphorylations in all isoforms. Phosphorylation at these serine residues previously has been shown to correlate with PKD activity [23], [24], [25].

We next tested if both PKD isoforms can interact as previously described by Bossard *et al*. [26]. Therefore, we first overexpressed FLAG-tagged PKD2 and GST-tagged PKD3, immunoprecipitated PKD2 (anti-FLAG) and detected PKD3 (anti-GST). We confirmed that PKD3 can be immunoprecipitated with PKD2 (**Fig. 1C**) and *vice versa* (not shown). To test if such complexes can be formed in cells by endogenous proteins we immunoprecipitated endogenous PKD2 or PKD3 using subtype specific antibodies, and then probed for the other isoform, respectively. Our data show that endogenous PKD2 and PKD3 also form a complex in both cell lines (**Fig. 1D**).

### Basal PKD3 Activity Mediates Basal Activity of the PAK4-LIMK-cofilin Pathway and Directed Cell Migration

**Figure 2:**
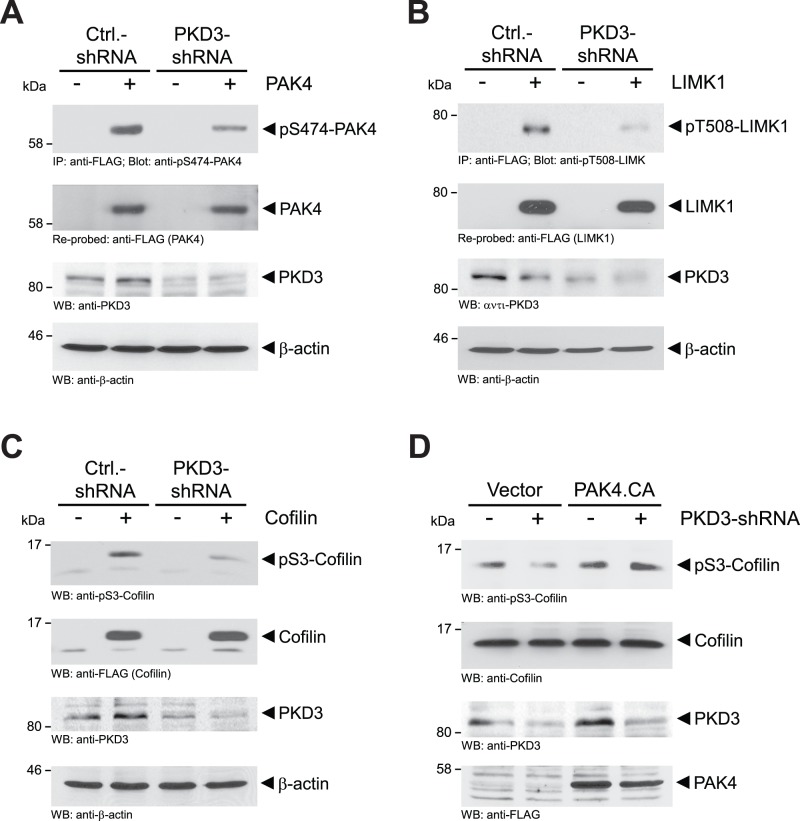
Basal PKD3 activity mediates constitutive activity of the PAK4-LIMK-cofilin pathway. **A, B:** HeLa cells were transfected with control shRNA (pSuper-scr-shRNA) or shRNA specifically-targeting PKD3 (pSuper-PKD3-shRNA) and next day also transfected with FLAG-tagged PAK4 (**A**) or LIMK1 (**B**), as indicated. 48 hours after initial infection, cells were lysed and PAK4 (**A**) or LIMK1 (**B**) was immunoprecipitated (anti-FLAG). Samples were subjected to SDS-PAGE, transferred to nitrocellulose and immunostained for PAK4 activity (anti-pS474-PAK4) (**A**) or LIMK1 activity (anti-pT508-LIMK1) (**B**). After stripping samples were re-probed with anti-FLAG for total PAK4 (**A**), or total LIMK1 (**B**). PKD3 knockdown was controlled by Western blotting (anti-PKD3) and equal loading was controlled by Western blotting for β-actin (anti-β-actin). **C:** HeLa cells were transfected with control shRNA (pSuper-scr-shRNA) or shRNA specifically-targeting PKD3 (pSuper-PKD3-shRNA) and next day also transfected with FLAG-tagged cofilin, as indicated. 48 hours after initial infection, cells were lysed, samples subjected to SDS-PAGE, transferred to nitrocellulose and immunostained for pS3-phosphorylated cofilin (anti-pS3-cofilin), cofilin (anti-FLAG), PKD3 knockdown (anti-PKD3) or β-actin (anti-β-actin) as loading control. **D:** HeLa cells were transfected with control shRNA (pSuper-scr-shRNA) or shRNA specifically-targeting PKD3 (pSuper-PKD3-shRNA) and next day also transfected with FLAG-tagged constitutively-active PAK4 (PAK4.CA), as indicated. 48 hours after initial infection, cells were lysed, samples subjected to SDS-PAGE, transferred to nitrocellulose and immunostained for pS3-phosphorylated cofilin (anti-pS3-cofilin), cofilin (anti-cofilin), PKD3 knockdown (anti-PKD3) or PAK4.CA (anti-FLAG). Experiments shown in A-D were performed at least three times with similar results.

Previously, we and others have shown that PKD isoforms can regulate cell migration by modulating cofilin activity via PAK4 and slingshot SSH1L [17], [18], [19], [20]. In HeLa and MDA-MB-468 cells endogenous PAK4 is basally-active as judged by its phosphorylation in the activation loop (**Fig. S1A**). We previously had shown that this residue can be targeted by PKD enzymes [19]. Therefore, we next tested if observed basal PKD3 activity in cells contributes to basal PAK4/LIMK signaling. When PKD3 was knocked-down using isoform-specific shRNA (PKD3-shRNA), PAK4 activity (judged by its phosphorylation status at the activation loop using the pS474-PAK4 antibody) was decreased (**Fig. 2A** for HeLa**,** and **Figure S1B** for MDA-MB-468). This also resulted in decreased phosphorylation of the PAK4 substrate LIMK1 (**Fig. 2B**), as well as decreased inactivation of cofilin by phosphorylation at its serine 3 residue (**Fig. 2C** for HeLa**,** and **Figure S1C** for MDA-MB-468). Next we tested if the effects of a knockdown of PKD3 on cofilin phosphorylation can be rescued with a constitutively-active PAK4 (PAK4.CA). This mutant of PAK4 has a serine to glutamate mutation at residue 474 (PAK4.S474E), mimicking the phosphorylated activation loop serine, and therefore is independent of regulation by PKD3 at this site. When transfected in PKD3-shRNA expressing cells constitutively-active PAK4 (PAK4.CA) restored phosphorylation of endogenous cofilin (**Fig. 2D**). This demonstrates that the decrease of cofilin phosphorylation that was observed when PKD3 is knocked-down is due to PKD3-mediated PAK4/LIMK signaling.

**Figure 3:**
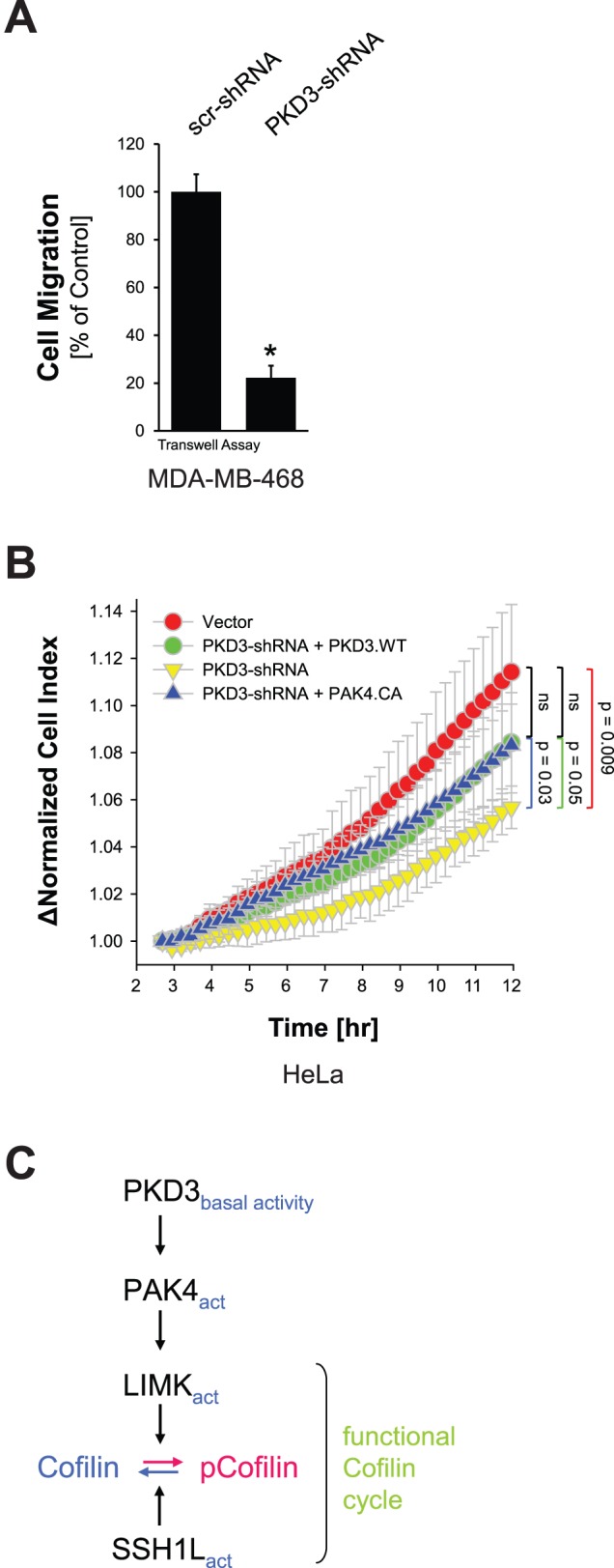
Basal PKD3-PAK4 signaling mediates basal directed cell migration. **A:** MDA-MB-468 cells were lentivirally-infected with control shRNA (scr-shRNA) or shRNA targeting the 3′-UTR of PKD3 (PKD3-shRNA). 48 hours after initial infection, a Transwell cell migration assay was performed and cell migration towards NIH-3T3-conditioned media was determined. Error bars represent three experiments. The asterisk indicates statistical significance. Control blots are shown in Figure S2A. **B:** HeLa cells were lentivirally-infected with control shRNA (scr-shRNA) or shRNA specifically-targeting the 3′-UTR of PKD3 (PKD3-shRNA) and next day transfected with vector control, wildtype PKD3 or PAK4.CA, as indicated. 48 hours after initial infection, cells were reseeded in Transwell CIM-plate 16 plates. After two hours of attachment, cell migration towards NIH-3T3-conditioned media over 10 hours was continuously monitored in real-time using the xCELLigence RTCA DP instrument. Error bars (grey) represent four experiments. Control blots are shown in Figure S2B. **C:** Scheme of how basal PKD3-PAK4 signaling mediates basal directed cell migration. Basal PKD3 activity activates LIMK via PAK4 leading to cofilin phosphorylation. This leads to a functional cofilin activity cycle and guarantees basal cell migration. Experiments shown in A-C were performed at least three times with similar results.

Since the PAK4/LIMK/cofilin signaling pathway is a key regulator of actin-driven cell motility, we next tested if PKD3 basal activity contributes to directed cell migration. The knockdown of basal PKD3 levels with a lentivirally-delivered shRNA targeting the 3′-UTR of PKD3 decreased basal cell migration in MDA-MB-468 cells (**Fig. 3A**, and control blots **Figure S2A**), and in HeLa cells (**Fig. 3B**, and control blots **Figure S2B**). This was partially rescued by expression of either wildtype PKD3, or constitutively-active PAK4 (PAK4.CA). This indicates that basal PKD3 activity under normal growth conditions of cells is contributing to a functional cofilin activity cycle and directed cell migration by activating PAK4 (**Fig. 3C**).

### RhoA Increases PKD2/3 Complex Formation and PKD2 and PKD3 Activities

**Figure 4:**
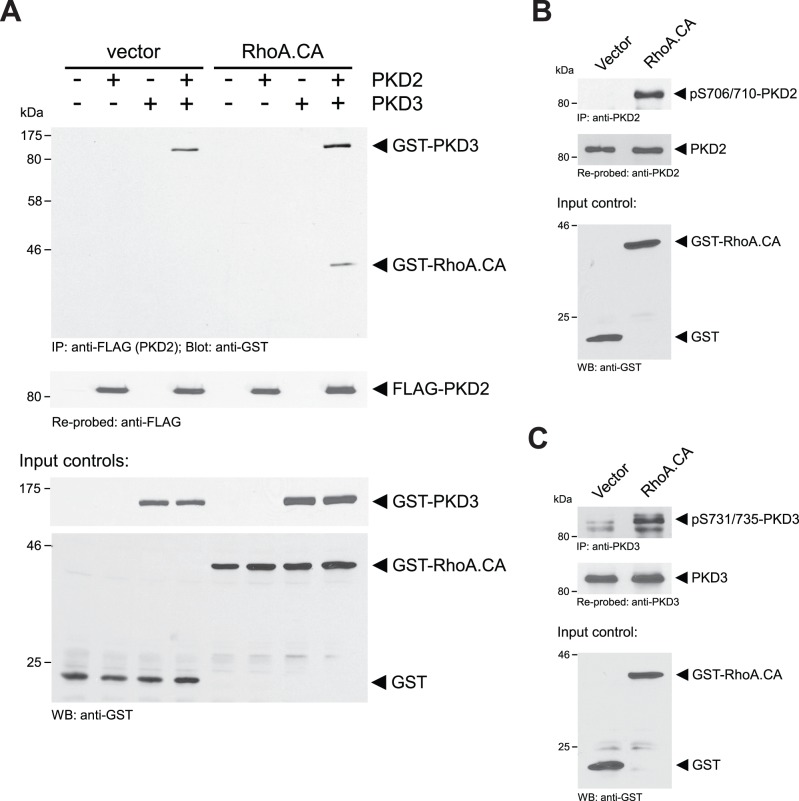
RhoA increases PKD2/3 complex formation and PKD2 and PKD3 activities. **A:** HeLa cells were transfected with vector control (pEBG; expresses GST), FLAG-tagged PKD2, GST-tagged PKD3 or GST-tagged active RhoA (pEBG-RhoA.CA), as indicated. Cells were lysed and PKD2 was immunoprecipitated (anti-FLAG). Samples were subjected to SDS-PAGE, transferred to nitrocellulose and immune-stained for co-immunoprecipitated PKD3 and RhoA.CA (anti-GST). After stripping samples were re-probed with anti-FLAG for PKD2. In addition, cell lysates were analyzed by Western blot (input control) for GST, GST-PKD3 or GST-RhoA.CA expression (anti-GST). **B, C:** HeLa cells were transfected with vector control (pEBG) or GST-tagged active RhoA (RhoA.CA), as indicated. Cells were lysed and endogenous PKD2 (**B**) or endogenous PKD3 (**C**) was immunoprecipitated (anti-PKD2 or anti-PKD3, respectively). Samples were subjected to SDS-PAGE, transferred to nitrocellulose and immunostained using anti-pS744/748 antibody that recognizes activation loop-phosphorylated PKD2 (pS706/710) or PKD3 (pS731/735). After stripping samples were re-probed with anti-PKD2 (**B**) or anti-PKD3 (**C**) antibodies. In addition, cell lysates were analyzed by Western blot (input control) for GST or GST-RhoA.CA expression (anti-GST). Experiments shown in A-C were performed at least three times with similar results.

We next tested the contribution of the PKD3 binding partner PKD2, which under normal growth conditions was not active (**Fig. 1B**), in this pathway. A typical activator for PKD enzymes that leads to modulation of the F-actin organization at the leading edge is the RhoGTPase RhoA [17], [27]. Activation of RhoA in cells increases stress fiber formation and decreases cell migration [28]. We first tested if active RhoA (RhoA.CA) impacts the formation of the PKD2/PKD3 complex. In presence of active RhoA increased binding of PKD3 to PKD2 was observed. In addition active RhoA itself was co-immunoprecipitated with PKD2 and PKD3 (**Fig. 4A**). We also found that active RhoA mediated activation of endogenous PKD2 (**Fig. 4B**), and also further increased the activity of endogenous PKD3 (**Fig. 4C**).

### Increased PKD2 and PKD3 Activities do not Further Enhance PAK4 Activity, but Inactivate SSH1L

**Figure 5:**
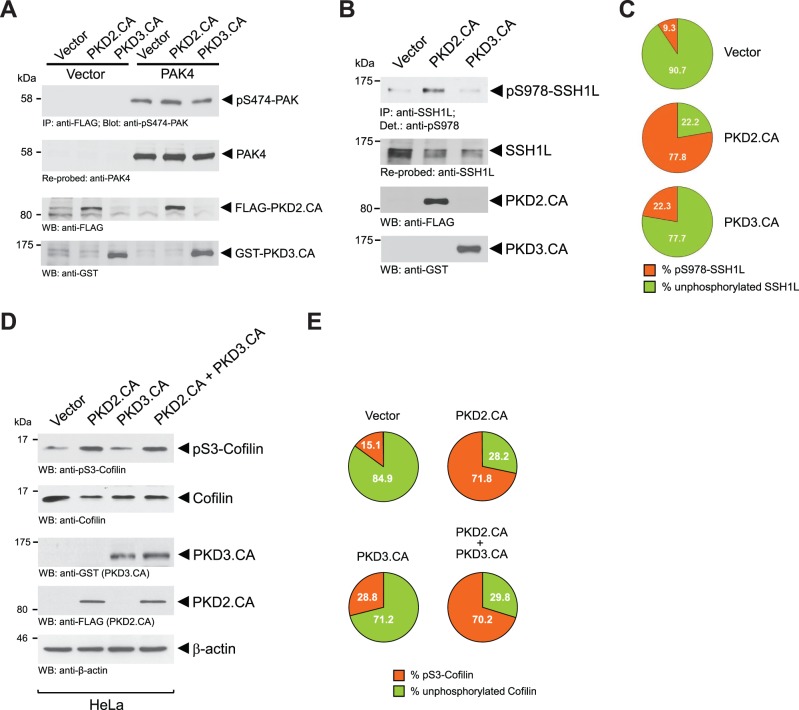
Increased PKD2 and PKD3 activities do not further enhance PAK4 activity, but inactivate SSH1L. **A:** HeLa cells were co-transfected with vector control or FLAG-tagged PAK4 and FLAG-tagged constitutively-active PKD2 or GST-tagged constitutively-active PKD3, as indicated. Cells were lysed, PAK4 was immunoprecipitated (anti-FLAG), samples subjected to SDS-PAGE, transferred to nitrocellulose and analyzed for PAK4 activity by immunostaining with anti-pS474-PAK4 antibody. After stripping samples were re-probed with anti-FLAG (total PAK4). In addition, cell lysates were analyzed by Western blot (input control) for expression of PKD2.CA (anti-FLAG) and PKD3.CA (anti-GST). **B:** HeLa cells were transfected with vector control, FLAG-tagged constitutively-active PKD2 or GST-tagged constitutively-active PKD3, as indicated. Cells were lysed, endogenous SSH1L was immunoprecipitated (anti-SSH1L), samples subjected to SDS-PAGE, transferred to nitrocellulose and analyzed for SSH1L phosphorylation at S978 by immunostaining with anti-pS978-SSH1L antibody. After stripping samples were re-probed with anti-SSH1L (total SSH1L). In addition, cell lysates were analyzed by Western blot (input control) for expression of PKD2.CA (anti-FLAG) and PKD3.CA (anti-GST). **C:** Percentage of S978-phosphorylated and unphosphorylated SSH1L was calculated and depicted in a pie graph. **D:** HeLa cells were transfected with control vector, FLAG-tagged constitutively-active PKD2 or GST-tagged constitutively-active PKD3, as indicated. Amount of endogenous pS3-phosphorylated cofilin and total cofilin was determined by Western blot analysis (anti-pS3-cofilin and anti-cofilin for total cofilin). **E:** Percentage of S3-phosphorylated and unphosphorylated cofilin was calculated and depicted in a pie graph. Experiments shown in A, B and C were performed at least three times with similar results.

We next tested how activation of PKD2 and further activation of PKD3 affects cofilin phosphorylation status and directed cell migration. Therefore, we made use of PKD plasmids that have mutations in their activation loop serines mimicking phosphorylation. These mutants represent constitutively-active alleles. Both, constitutively-active PKD2 (PKD2.CA; PKD2.S706E.S710E) and PKD3 (PKD3.CA; PKD3.S731E.S735E), did not further activate PAK4, indicating that this kinase is already at maximum activity under normal growth conditions (**Fig. 5A**).

PKD-mediated phosphorylation of overexpressed SSH1L at S978 previously had been shown to lead to its inactivation, with the net effect of increasing phospho-cofilin levels [17], [18], [19]. However, at the level of endogenous SSH1L a difference between both PKD isoform was detected (**Fig. 5B**). While active PKD2 significantly increased phosphorylation of SSH1L at S978, active PKD3 led to a significantly smaller increase of phosphorylation at this site (**Fig. 5C**). These data indicate that out of the two PKD isoforms expressed in HeLa, PKD2 is the main regulator of endogenous SSH1L. Overall, expression of active PKD2 dramatically increased phospho-cofilin levels from 15.1 percent in the vector control to 71.8 percent (**Figs. 5D** and **5E**). Expression of active PKD3, probably due to its weaker effects on inhibition of SSH1L, also led to an increase of cofilin phosphorylation, but only to 28.8 percent. A combination of both, active PKD2 and PKD3 did not top the effects on cofilin phosphorylation observed by active PKD2 alone. This is not surprising since both kinases can target SSH1L via the same phosphorylation site and additive effects are not expected.

### Net Effects of PKD Activities above Basal Levels are Loss of Barbed End Formation and Decreased Directed Cell Migration

**Figure 6:**
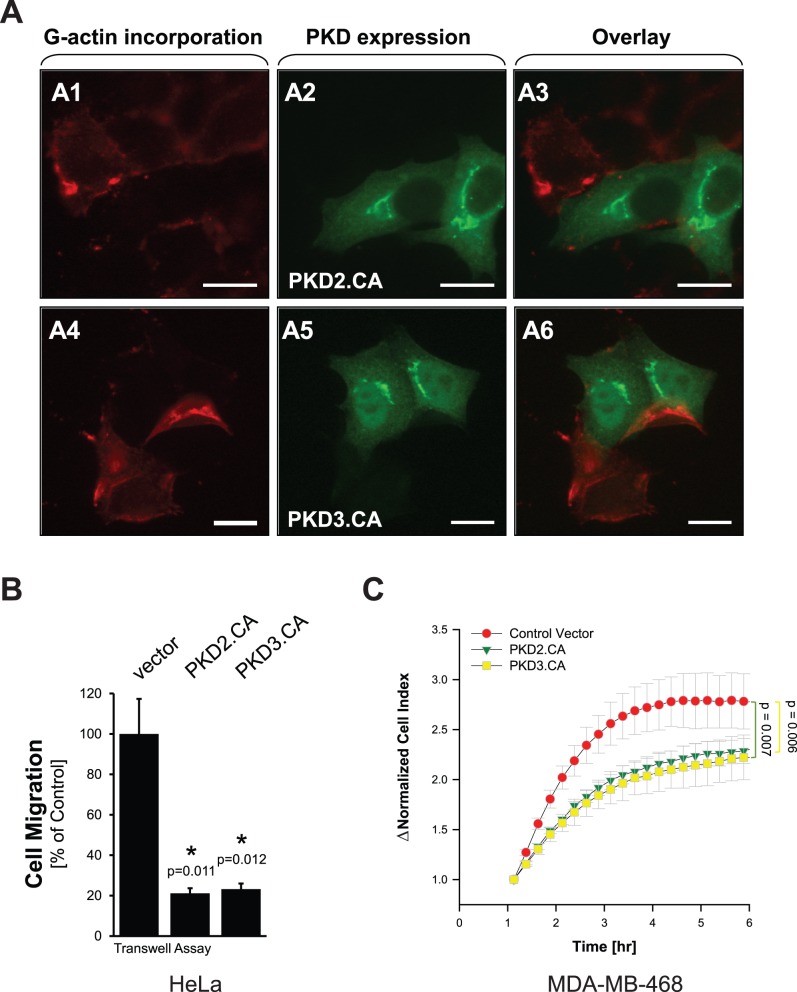
Net effects of increased PKD activities above basal levels are loss of barbed end formation and decreased directed cell migration. **A:** HeLa cells were transfected with FLAG-tagged constitutively-active PKD2 or GST-tagged constitutively-active PKD3. Next day, barbed end assay was performed as described in materials and methods. PKD2.CA or PKD3.CA-expressing cells were stained by immunofluorescence using primary antibodies directed against their tag as well as Alexa Fluor 488 as secondary antibody. The bar is 20 µm. The experiment was performed three times with similar results. **B:** HeLa cells were transfected with control vector, FLAG-tagged constitutively-active PKD2 or GST-tagged constitutively-active PKD3. 24 hours after initial infection, a Transwell cell migration assay was performed and cell migration towards NIH-3T3-conditioned media was determined. Error bars represent three experiments. The asterisk indicates statistical significance. Control blots are shown in Figure S3A. **C:** MDA-MB-468 cells were transfected with control vector, FLAG-tagged constitutively-active PKD2 or GST-tagged constitutively-active PKD3. 24 hours after initial transfection, cells were reseeded in Transwell CIM-plate 16 plates. After one hour of attachment, cell migration towards NIH-3T3-conditioned media over 5 hours was continuously monitored in real-time using the xCELLigence RTCA DP instrument. Error bars (grey) represent four experiments. P values indicate statistical significance. Control blots are shown in Figure S3B.

We next tested if the effects on cofilin phosphorylation observed when PKD2 and PKD3 are constitutively-active directly lead to decreased G-actin incorporation and to altered cell migration. Barbed end formation and G-actin incorporation in cells expressing active PKD2 or PKD3 was significantly decreased, indicating reduced cofilin activity (**Fig. 6A, A** **1–A6**) as compared to non-transfected cells on the same slide. The effects of both constitutively-active PKD isoforms on barbed end formation and G-actin incorporation directly corresponded with their effects on cell migration of HeLa and MDA-MB-468 cells (**Figs. 6B, 6C** and control blots **Figure S3A, S3B**).

**Figure 7:**
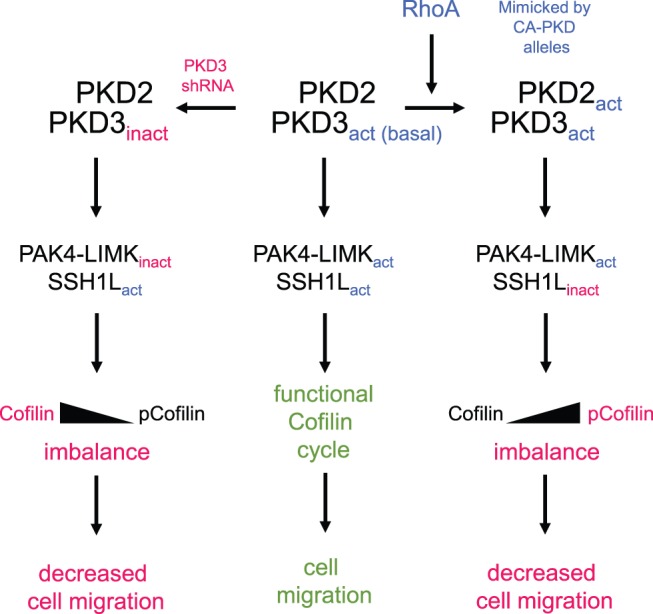
Scheme of how PKD subtypes may contribute to regulate cofilin activity and directed cell migration. Using HeLa cells, which only express the PKD subtypes PKD2 and PKD3 as a model system, we suggest the following scheme: In cells under normal growth conditions (middle), PKD2 and PKD3 form a complex in which PKD3 is basally active. Basal activity of PKD3 contributes to PAK4/LIMK activity. Together with active (unphosphorylated) SSH1L, active PAK4 guarantees a functional cofilin activity cycle and cell migration. If PKD3 is inactivated or decreased in its expression (left side), PAK4 activity decreases, while SSH1L stays active. Net effect is an increase in non-phosphorylated (active) cofilin. This leads to imbalanced F-actin severing and decreased cell migration. On the other hand, when PKD2 and PKD3 are further activated (i.e. by activation of RhoA) they phosphorylate SSH1L at S978, which leads to its inactivation (right side). In this scenario, basal PAK4 activity and decreased SSH1L activity increase phospho-cofilin levels dramatically, preventing F-actin severing and directed cell migration.

Taken together, by using HeLa and MDA-MB-468 cells as model systems to understand the roles of PKD isoform complexes on actin cytoskeleton-driven directed cell migration, we suggest the following mechanism: In cells under normal growth conditions (**Fig. 7** **, middle**), PKD2 and PKD3 form a complex in which PKD3 is basally active. Active PKD3 contributes to PAK4/LIMK activity. Together with active SSH1L, active PAK4 guarantees a functional cofilin activity cycle and cell migration. If PKD3 is inactivated (**Fig. 7** **, left side**), PAK4 activity decreases, while SSH1L stays active. Net effect is an increase in non-phosphorylated (active) cofilin. This leads to imbalanced F-actin severing and decreased cell migration. On the other hand, when PKD2 and PKD3 are further activated (i.e. mimicked by expression of a constitutive active version; or induced by active RhoA) they phosphorylate SSH1L at S978, which leads to its inactivation (**Fig. 7** **, right side**). Basal PAK4 activity in cells as well as decreased SSH1L activity in this scenario increases phospho-cofilin levels dramatically. Decreased cofilin activity prevents F-actin severing and also decreases directed cell migration.

## Discussion

Cell motility is mediated by dynamic actin remodeling at the leading edge [29]. A key enzyme in mediating actin remodeling in migrating cells is cofilin [4]. In order for cofilin to mediate actin reorganization processes it needs to undergo cyclic phosphorylation and dephosphorylation events (cofilin cycle) [3] and both hyper- and hypo-phosphorylation of the total cellular cofilin pool can lead to decreased directed cell migration. Cofilin activity is regulated by its phosphorylation status at serine residue S3. Phosphorylation is mediated by LIMK1/2 [3], [30], [31] and renders the enzyme inactive. Phosphatases such as SSH1L dephosphorylate cofilin at S3 and re-enter it into the active pool [7], [32]. While SSH1L often is constitutively-active in cells, LIMK1/2 activity is regulated through direct phosphorylation of its activation loop at T508, which can be mediated by group II PAK kinases [3], [33]. Of these, PAK4 and PAK5 have essential roles in controlling cell motility since they locate to the leading edge (reviewed in [34]). In many cancer cell lines PAK4 was shown to be constitutively-active [34], and PAK4 and other members of this kinase family emerge as targets for the development of inhibitors for clinical use [34].

Protein Kinase D (PKD) isoforms are regulators of cofilin-driven actin reorganization and directed cell migration through both, p21-activated kinase 4 (PAK4) and the phosphatase slingshot 1L (SSH1L) [12], [16]. The relative contributions of different endogenous PKD isoforms to both signaling pathways have not been fully elucidated. Puzzling in the PKD literature is that different isoforms, or even the same isoform, have been attributed pro- or anti-migratory functions [12], [14], [17], [18], [19], [20], [21], [22], [35], [36], [37], [38], [39], [40], [41], [42], [43], [44]. For example, for PKD3 it was shown that chemical inhibition [22], but also overexpression of active PKD3 blocked directed cell migration [19]. With the data presented here as well as the scheme suggested (**Fig. 7**), we provide an explanation for these, on the first few conflicting results. We show that similar as it was suggested for LIMK before [1], [45], level and activity of PKD enzymes regulate the cofilin phosphorylation status. Both, decreased basal activity of PKD3 (resulting in decreased PAK4 activity and cofilin hypo-phosphorylation), or increased activities of PKD2 and PKD3 (resulting in additional inhibition of SSH1L and cofilin hyper-phosphorylation), promote that the cofilin activity cycle is dysfunctional and directed cell migration is decreased (**Fig. 7**). Overall our data indicate that PKD enzymes, dependent on activity level and stimulus, can modulate the cell’s migration status.

Another level of complexity in regulation of cofilin activity is achieved because PKD enzymes can form protein complexes (**Fig. 1** and [26]). To simplify our studies we used two cell lines as a model system that only express PKD2 and PKD3. Of the group II PAK kinases HeLa also mainly express PAK4 and no/or marginal levels of PAK5 and PAK6 [19]. We found that in both cell lines under normal growth conditions PKD2 and PKD3 can form complexes, in which PKD3 is basally active and PKD2 is inactive (**Fig. 1**). Basal activity of PKD3 mediates PAK4 activity and downstream signaling (**Fig. 2**) while SSH1L under these growth conditions is active [17]. Overall this signaling constellation occurring under normal growth conditions seems to guarantee a functional cofilin phosphorylation cycle facilitating directed cell migration. This is supported by our data showing that under normal growth conditions a knockdown of PKD3 decreases the activity of the PAK4/LIMK signaling cascade and results in hypo-phosphorylation of cofilin (**Fig. 2**), with the net effect of a decrease of directed cell migration (**Fig. 3**). Re-expression of active PAK4 in PKD3 knockdown cells, although it fully restored phospho-cofilin levels (**Fig. 2D**), only led to a partial rescue of directed cell migration (**Fig. 3**). One explanation for this may be that PKD3 not only regulates PAK4 activity, but also its translocation to the F-actin structures at the leading edge [46]. Due to the knockdown of PKD3, not all of the PAK4.CA may be localized at the leading edge. Therefore, although fully active towards cofilin, PAK4.CA only partially restores cell migration.

While under normal growth conditions in HeLa and MDA-MB-468 cells basal PKD3 activity facilitates a normal cofilin phosphorylation and dephosphorylation cycle that is necessary for directed cell migration, further activation of PKD enzymes (PKD2 and PKD3 in the investigated cell lines) also can cause a decrease in directed cell migration. On the first view this seems paradox to effects obtained when PKD3 is knocked down, but can be explained by our data. Regulators and inducers of PKD activity are RhoGTPases including Rac1, RhoA and Cdc42 [14], [17], [20], [27], [47], [48]. Here we mainly focused on activation of PKD enzymes by RhoA, since active RhoA increases F-actin filament formation and decreases cell migration [28], [49]. In HeLa, we found that a constitutively-active allele of RhoA induced PKD2 activity and further increased PKD3 activity (**Fig. 4**). To mimic of activation PKD2 and PKD3 and to investigate the role of full PKD activity on directed cell migration, we utilized constitutively-active mutants. We found that activation of PKD2 and (to some extend) further activation of PKD3 leads to increased phosphorylation of SSH1L at S978 (**Fig. 5C**). Phosphorylation at this site previously was shown to decrease its localization with F-actin filaments to facilitate binding to 14-3-3 proteins and sequestration in the cytosol [17], [18], [20], [50]. Net effect of both active PKD3 (increased PAK4 activity) and PKD2 (decreased SSH1L activity) is a dramatic increase in phospho-cofilin levels. This imbalance in the cofilin phosphorylation and dephosphorylation cycle also translates to decreased directed cell migration, since now both cofilin regulatory pathways are controlled by the active PKD2/PKD3 complex (**Figs. 5D, 5E** and **6**).

In summary, our data allow to explain the paradox previously noted by multiple laboratories, describing that either increased activation, or inactivation of PKD proteins can have similar effects on cell migration. Our data suggest that PKD enzymes are important regulators of directed cell migration by providing an interface for both cofilin regulatory pathways. Dependent on the activity of involved PKD enzymes signaling can be balanced to guarantee a functional cofilin activity cycle and increase cell migration, or imbalanced to decrease cell migration.

## Materials and Methods

### Cell Lines, Antibodies, and Reagents

HeLa cells were obtained from ATCC and maintained in DMEM with 10% FBS. Anti-GST antibody was from Santa Cruz (Santa Cruz, CA), anti-HA, anti-FLAG (M2) and anti-β-actin from Sigma-Aldrich (St Louis, MO), anti-GFP/YFP (Ab290) from Abcam (Cambridge, MA), anti-PKD3 and anti-SSH1L from Bethyl Laboratories (Montgomery, TX), anti-pS744/748-PKD (recognizes pS706/710 for human PKD2 and pS731/735 for human PKD3), anti-LIMK, anti-pT508-LIMK, anti-PAK4, anti-pS474-PAK4, anti-Cofilin and anti-pS3-Cofilin from Cell Signaling Technology (Danvers, MA), anti-PKD2 from Millipore (Billerica, MA). The monoclonal antibodies directed against PKD1 and pS978-SSH1L were described and characterized previously [20]. Secondary HRP-linked antibodies were from Millipore. Secondary antibodies for immunofluorescence analysis were goat-anti-mouse Alexa Fluor 488 F(ab’)2 and goat-anti-rabbit Alexa Fluor 488 F(ab’)2 from Invitrogen (Carlsbad, CA). TransIT HeLa-Monster (Mirus, Madison, WI) was used for transient transfection of cells.

### DNA Constructs

The expression constructs for GST-tagged PKD3, GST- tagged PKD3.CA, FLAG-tagged PKD2, FLAG-tagged PKD2.CA, GST-tagged RhoA.CA, FLAG-tagged cofilin, YFP-tagged cofilin, FLAG-tagged LIMK and FLAG-tagged human PAK4 were described previously [17], [19], [50], [51], [52]. To obtain a constitutively-active PAK4.S474E mutant (PAK4.CA), site-directed mutagenesis was carried out using the QuikChange kit (Stratagene, La Jolla, CA), the FLAG-tagged PAK4 expression construct as template and the following primer pair: 5′- GAAGTGCCCCGAAGGAAGGAGCTGGTCGGCACGCCCTAC-3′ and 5′-GTAGGGCGTGCCGACCAGCTCCTTCCTTCGGGGCACTTC-3′. pSuper-PKD3-shRNA (used in Figure 2) was obtained by cloning the following oligonucleotides into pSuper: 5′-GATCCCCGACGGGACTCTCTGCCCGATTCAAGAGATCGGGCAGAGAGTCCCGTCTTTTTGGAAA-3′ and 5′-AGCTTTTCCAAAAAGACGGGACTCTCTGCCCGATCTCTTGAATCGGGCAGAGAGTCCCGTCGGG-3′. Lentiviral shRNA directed against human 3′-UTR of PKD3 (1 NM_005813.x-3393s1c1 shRNA, target sequence: CGGGAAACTGAATAATAAGAA) was obtained from Sigma.

### Immunoblotting, Immunoprecipitation and PAGE

Cells were washed twice with ice-cold PBS (140 mM NaCl, 2.7 mM KCl, 8 mM Na_2_HPO_4_, 1.5 mM KH_2_PO_4_, pH 7.2) and lysed in buffer A (50 mM Tris-HCl pH7.4, 1% Triton X-100, 150 mM NaCl, 5 mM EDTA pH 7.4) plus Protease Inhibitor Cocktail (PIC, Sigma-Aldrich). Lysates were vortexed, incubated on ice for 30 minutes and centrifuged (13,000 rpm, 15 min, 4°C). Protein concentration of lysates was determined and lysates were subjected to Western blot analysis, or proteins of interest were immunoprecipitated by 1 hour incubation with a specific antibody (2 µg) followed by 30 minutes incubation with protein G-Sepharose (Amersham Biosciences). Immunecomplexes were washed 3 times with TBS (50 mM Tris-HCl pH 7.4, 150 mM NaCl) and resolved in 20 µl TBS plus 2x Laemmli buffer. Samples were subjected to SDS-PAGE, transferred to nitrocellulose membranes and visualized by immunostaining.

### Analysis of Free Actin Filament Barbed Ends

Cells were transfected as indicated and reseeded on 8 well ibiTreat µ-Slides (Integrated BioDiagnostics, Martinsried, Germany). Cells were serum-starved for 16 hours. The medium was removed, cells were washed with pre-warmed PBS, permeabilized and labeled with 0.4 µM actin from rabbit muscle conjugated to Alexa Flour 568 in permeabilization buffer (20 mM HEPES, 138 mM KCl, 4 mM MgCl_2_, 3 mM EGTA, 0.2 mg/ml saponin, 1% BSA) plus 1 mM ATP for 15 seconds at 37°C. The cells were fixed with 4% paraformaldehyde in PBS (RT, 10 min), and samples were examined using a IX81 DSU Spinning Disc Confocal from Olympus.

### Reverse Transcriptase-Polymerase Chain Reaction (RT-PCR)

Cellular RNA was isolated using RNA-Bee (TEL-TEST, Friendswood, TX) according to the manufacturer’s instructions and mRNA was transcribed into cDNA using Superscript II (Invitrogen). For the transcription reaction 1 µg Oligo dT(18) primer (New England Biolabs, Beverly, MA) and 1 µg RNA were incubated in a total volume of 10 µl H_2_O at 70°C for 10 min. 5x buffer, 40 U RNAsin (Roche, Mannheim, Germany), 200 µM dNTP (NEB), 10 mM DTT, 300 U Superscript II reverse transcriptase were added to a total volume of 20 µl. The reaction was carried out at 45°C for 60 minutes and then heat-inactivated at 95°C for 5 minutes. Resulting cDNA was subjected to PCR analysis using specific primer sets. Primers for a 600 bp fragment of human PKD1 were 5′-TTCTTCCACCTCAGGTCATC-3′ and 5′-TGCCAGAGCACATAACGAAG-3′, a 698 bp fragment of PKD2 were 5′-CAACCCACACTGCTTTGAGA-3′ and 5′-CACACAGCTTCACCTGAGGA-3′, and a 485 bp fragment of PKD3 were 5′-TCATTGACAAACTGCGCTTC-3′ and 5′-GTACATGATCACGCCCACTG-3′. Reaction conditions for the PCR reactions were: 1 min annealing at 55°C, 1 min amplification at 72°C, with 25 cycles.

### Cell Migration Assays

Transwell migration assays were performed as previously described [17], [19]. To measure impedance-based real-time cell migration, cells were transfected with plasmids of interest as indicated. 24 hours (overexpression) or 48 hours (shRNA) after transfection, cells were re-seeded on Transwell CIM-plate 16 plates from Roche (Indianapolis, IN). After attachment of cells (3 hours after re-seeding for HeLa, 1 hour for MDA-MB-468), cell migration towards NIH-3T3-conditioned media was continuously monitored in real-time for indicated hours) using the xCELLigence RTCA DP instrument (Roche). Error bars (grey) represent three experiments.

## Supporting Information

Figure S1 **A: HeLa or MDA-MB-468 cells were analyzed for activity of endogenous PAK4.** Endogenous PAK4 was immunoprecipitated. Samples were subjected to SDS-PAGE and analyzed for PAK4 activity by immunoblotting with anti-pS474 antibody (recognizes the phosphorylated activation loop of PAK4). Samples were then re-probed for PAK4. **B:** MDA-MB-468 cells were lentivirally-infected with control shRNA (or shRNA specifically-targeting PKD3 and next day also transfected with FLAG-tagged PAK4, as indicated. 48 hours after initial infection, cells were lysed and PAK4 was immunoprecipitated (anti-FLAG). Samples were subjected to SDS-PAGE, transferred to nitrocellulose and immunostained for PAK4 activity (anti-pS474). After stripping samples were re-probed with anti-FLAG for total PAK4. PKD3 knockdown was controlled by Western blotting (anti-PKD3) and equal loading was controlled by Western blotting for β-actin (anti-β-actin). **C:** MDA-MB-468 cells were lentivirally-infected with control shRNA or shRNA specifically-targeting PKD3 and next day also transfected with FLAG-tagged cofilin, as indicated. 48 hours after initial infection, cells were lysed, samples subjected to SDS-PAGE, transferred to nitrocellulose and immunostained for pS3-phosphorylated cofilin (anti-pS3-cofilin), cofilin (anti-FLAG), PKD3 knockdown (anti-PKD3) or β-actin (anti-β-actin; loading control).
(PDF)

Figure S2 **A: Control blots for** **Figure 3A** **.** Lysates were analyzed for expression of endogenous PKD3 (Western blot: anti-PKD3). Western blots for β-actin (anti-β-actin) served as loading control. **B:** Control blots for Figure 3B. Lysates were analyzed for expression of endogenous PKD3 (Western blot: anti-PKD3), ectopically-expressed PKD3 (Western blot: anti-GST), or ectopically-expressed PAK4.CA (Western blot: anti-FLAG). Western blots for β-actin (anti-β-actin) served as loading control.
(PDF)

Figure S3 **A: Control blots for** **Figure 6B** **.** Lysates were analyzed for expression of PKD2.CA (Western blot: anti-FLAG), or PKD3.CA (anti-GST). Western blots for β-actin (anti-β-actin) served as loading control. **B:** Control blots for Figure 6C. Lysates were analyzed for expression of PKD2.CA (Western blot: anti-FLAG), or PKD3.CA (anti-GST). Western blots for β-actin (anti-β-actin) served as loading control.
(PDF)
