# Supplementary figures and images for: Protein Kinase D Isoforms Differentially Modulate Cofilin-Driven Directed Cell Migration
Source: PLoS One. 2014 May 19;9(5):e98090. doi: 10.1371/journal.pone.0098090 (PMC4026536; doi:10.1371/journal.pone.0098090)

Supplemental Figure S1

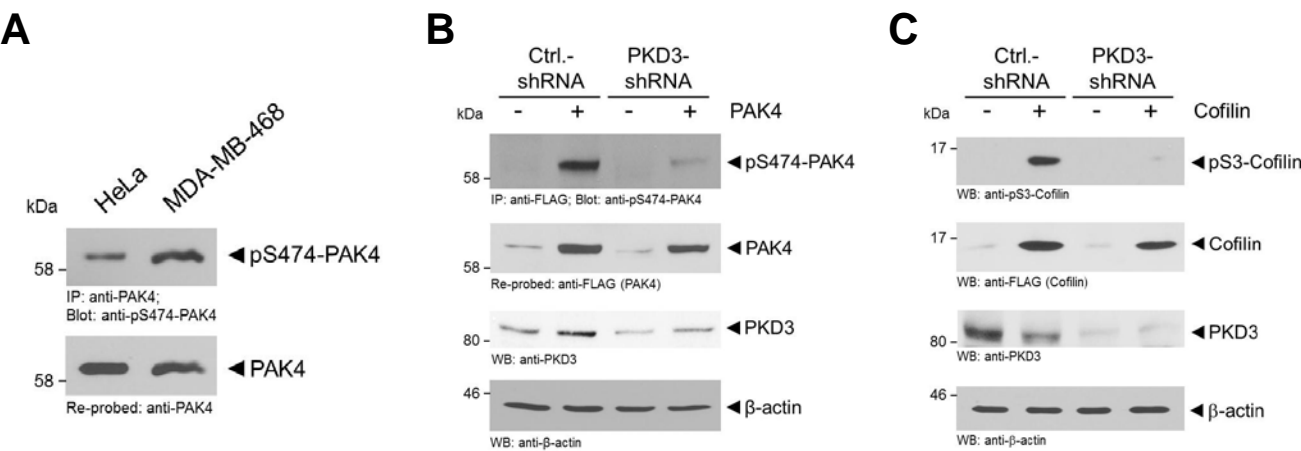

Supplement: Figure S1 — A: HeLa or MDA-MB-468 cells were analyzed for activity of endogenous PAK4. Endogenous PAK4 was immunoprecipitated. Samples were subjected to SDS-PAGE and analyzed for PAK4 activity by immunoblotting with anti-pS474 antibody (recognizes the phosphorylated activation loop of PAK4). Samples were then re-probed for PAK4. B: MDA-MB-468 cells were lentivirally-infected with control shRNA (or shRNA specifically-targeting PKD3 and next day also transfected with FLAG-tagged PAK4, as indicated. 48 hours after initial infection, cells were lysed and PAK4 was immunoprecipitated (anti-FLAG). Samples were subjected to SDS-PAGE, transferred to nitrocellulose and immunostained for PAK4 activity (anti-pS474). After stripping samples were re-probed with anti-FLAG for total PAK4. PKD3 knockdown was controlled by Western blotting (anti-PKD3) and equal loading was controlled by Western blotting for β-actin (anti-β-actin). C: MDA-MB-468 cells were lentivirally-infected with control shRNA or shRNA specifically-targeting PKD3 and next day also transfected with FLAG-tagged cofilin, as indicated. 48 hours after initial infection, cells were lysed, samples subjected to SDS-PAGE, transferred to nitrocellulose and immunostained for pS3-phosphorylated cofilin (anti-pS3-cofilin), cofilin (anti-FLAG), PKD3 knockdown (anti-PKD3) or β-actin (anti-β-actin; loading control). (PDF) [file pone.0098090.s001.pdf]

**A**

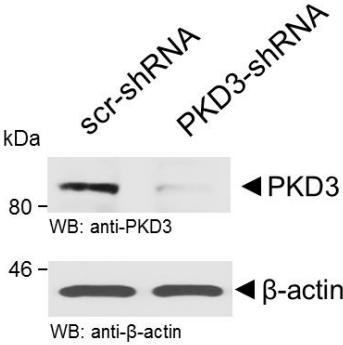

**B**

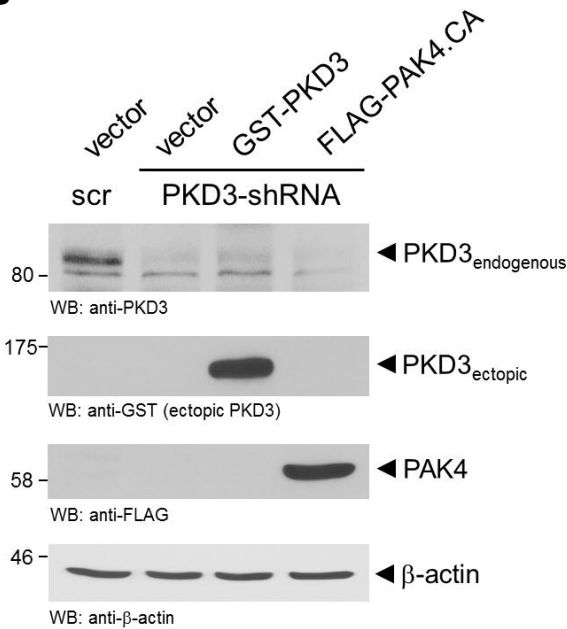

Supplement: Figure S2 — A: Control blots for Figure 3A . Lysates were analyzed for expression of endogenous PKD3 (Western blot: anti-PKD3). Western blots for β-actin (anti-β-actin) served as loading control. B: Control blots for Figure 3B. Lysates were analyzed for expression of endogenous PKD3 (Western blot: anti-PKD3), ectopically-expressed PKD3 (Western blot: anti-GST), or ectopically-expressed PAK4.CA (Western blot: anti-FLAG). Western blots for β-actin (anti-β-actin) served as loading control. (PDF) [file pone.0098090.s002.pdf]

Supplemental Figure S3

**A**

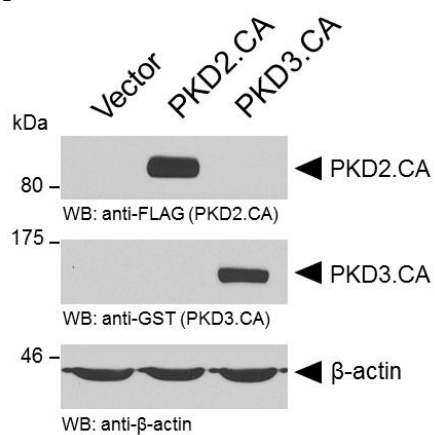

**B**

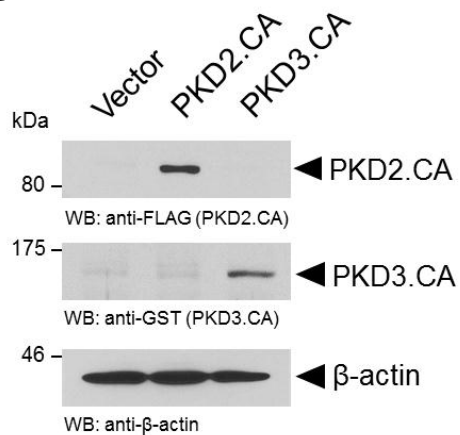

Supplement: Figure S3 — A: Control blots for Figure 6B . Lysates were analyzed for expression of PKD2.CA (Western blot: anti-FLAG), or PKD3.CA (anti-GST). Western blots for β-actin (anti-β-actin) served as loading control. B: Control blots for Figure 6C. Lysates were analyzed for expression of PKD2.CA (Western blot: anti-FLAG), or PKD3.CA (anti-GST). Western blots for β-actin (anti-β-actin) served as loading control. (PDF) [file pone.0098090.s003.pdf]
